# Expression of adhesion molecules on granulocytes and monocytes following myocardial infarction in rats drinking white wine

**DOI:** 10.1371/journal.pone.0196842

**Published:** 2018-05-10

**Authors:** Nikolina Režić-Mužinić, Angela Mastelić, Benjamin Benzon, Anita Markotić, Ivana Mudnić, Ivica Grković, Mia Grga, Ana Marija Milat, Nikola Ključević, Mladen Boban

**Affiliations:** 1 Department of Medical Chemistry and Biochemistry, University of Split School of Medicine, Split, Croatia; 2 Department of Anatomy, Histology and Embryology, University of Split School of Medicine, Split, Croatia; 3 Department of Pharmacology, University of Split School of Medicine, Split, Croatia; University of Alabama at Birmingham, UNITED STATES

## Abstract

Neutrophils and monocytes through their CD15s, CD11b and CD44 adhesion molecules are implicated in the initiation and resolution of cardiac inflammation as well as in healing processes after the myocardial infarction (MI). The aim of this study was to determine the effect of white wine consumption on granulocyte and monocyte CD15s, CD11b, and CD44 expression 24h after the surgically inflicted MI. Granulocytes and monocytes were analyzed by flow cytometry, using whole blood of male Sprague–Dawley rats that consumed white wine for 4 weeks. This group was compared with water only drinking controls, sham animals (subject to surgery without myocardial infarction) and baseline group (intact animals that received no intervention prior to being sacrificed). Sham animals did not differ from baseline animals in CD11b+CD44+ percentage and CD44+ median fluorescence intensity. Wine drinking was associated with striking increase in CD44 expression on monocyte subpopulations. Its expression was three and fourfold increased on monocytes and large monocytes, respectively, relative to the water only drinking controls. Because of known role of CD44 on suppression of post-infarction inflammation, its upregulation on granulocytes and monocytes may significantly contribute to the microenvironment favourable for the cardiac regeneration.

## Introduction

An inflammatory response to the myocardial infarction (MI) is a prerequisite for optimal healing and scar formation [1]. Infarct healing largely depends on neutrophil and monocyte infiltration and clearance of the injured tissue from dead cells and matrix debris [2]. Neutrophil transmigration into the injured myocardium is a result of their interactions with the endothelium [1]. Endothelial selectin binding to CD15s neutrophil glycoantigen mediates the initial tethering of neutrophils to the endothelial cell surface. This enables leukocytes to roll along the venular wall and to interact with activating factors leading to neutrophil integrin (such as CD11b) activation [1]. Binding of neutrophil CD11b to Intercellular Adhesion Molecule 1(ICAM-1) [3], expressed on stimulated endothelial cells, leads to firm adhesion of leukocytes [1]. The same adhesion molecules are implicated in monocyte extravasation [4]. After passing the endothelial barrier, neutrophil and monocyte CD44 receptor interacts with hyaluronate in the extracellular matrix [5–7]. Hyaluronate participates in organization of the extracellular matrix and is increased during inflammation and tissue remodelling [8, 9]. CD44 is crucial for optimal infarct healing since it regulates post-infarction cardiac remodelling [10]. Accentuation, prolongation or expansion of the post-infarction inflammatory response may result in unfavourable remodelling and myocardial dysfunction following the MI [2].

Based on the epidemiological evidence of its beneficial effects on cardiovascular health, red wine and its constituents were used in many studies examining their potential to protect myocardium against the ischemic injury [11]. Different favourable cardiovascular endpoints are attributed to the phenolic compounds found in red wine [12] [13], although their exact mechanisms of action are still debatable [14].

In contrast to red wines, in a standard white wine production, the grape juice is fermented without seeds and skins, which are most important sources of phenolic compounds, resulting with much lower total phenolic content in white wines [15]. So, biological potentials of white wines may considerably differ from that of red wines. However, strong epidemiological data demonstrating superiority of red over white wines are still missing since *in vivo* studies examining biological effects of white wine are rather scarce [16–18].

Therefore, we performed a four weeks consumption trial with white wine evaluating the effects on granulocyte and monocyte inflammatory markers (CD15s, CD11b and CD44), during inflammatory phase following experimental myocardial infarction in rats.

## Materials and methods

All rats were raised under controlled conditions (temperature, 22±2°C; light schedule, 14 h of light and 10 h of dark) at the University of Split Animal Facility. Animals were fed with standard rat chow Mucedola 3.9 kcal/g *ad libitum* (Settimo Milanese, Milan, Italy). Care of animals was carried out following the “Guide for the care and use of laboratory animals” (1985); NIH, Bethesda and was approved both at institutional level by the University of Split, Medical School Ethics Committee (Document No. 2181-198-03-04-13-0042) and at national level by the Ministry of Agriculture, Directorate for Veterinary and Food Safety (Document No. 525-10/0255-16-7).

### Study design

One month old Sprague-Dawley male rats were randomized into four groups: animals drinking wine and water for 28 days (group W, n = 9), animals drinking water only (control group, C, n = 9), the sham group (group SH, n = 9) and the baseline group (group BL, n = 11). In the wine group, for four weeks prior the surgical intervention, the animals consumed white wine Graševina Krauthaker vintage 2015, containing 13.0% alcohol, at an average dosage of 5 mL/100g body weight.

Our calculations, which are a subject of a separate study [19], show that the total daily intake of alcohol (of about 1 gram per day), and its contribution to the daily caloric intake is well within the moderate drinking pattern, when translated to human scenarios. Baseline group included intact animals that received no intervention prior to being sacrificed.

Rats where anesthetized with a mixture of Ketaminol (Ketaminol 10, 1.2 ml/kg, Intervet International, Netherlands) and Xylazinum (Xylapan, 0.4 ml/kg, Vetoquintol, Switzerland) injected in the right hamstring muscles. The surgical procedure was performed using the ‘abdominal approach’ to the heart, as previously described [20]. Briefly, with the aid of a surgical microscope (Leica, M520 MC1, Switzerland), a midline incision of the anterior half of the diaphragm was performed while the animal was connected to a ventilator (SAR 830, CWE Inc, USA) set on 59 breaths/min. The left anterior descending artery was identified on the anterior surface of the heart and ligated (with a 7.0 nonresorbable suture) about 2 mm from the auricular margin. In sham animals, the needle was passed under the artery, but ligation was not performed. Immediate colour change of the left ventricular surface (pale appearance) extending to the apex of the heart was a sign of successful coronary ligation.

After the air was expelled from the thoracic cavity following a surgical repair of the diaphragm, animals were removed from the ventilator and resumed spontaneous respirations.

Following performance of coronary ligation in W and C groups, animals were left to survive for 24 hours and then sacrificed in full anaesthesia (the above described combination of anaesthetics) by decapitation.

The presence of the infarction was confirmed by finding a zone of complete ischemia in the anterior wall of the left ventricle on cross sections (absence of blood cells infiltration). The peri-infarct area was found on the other side of the ischemic zone and was characterised by hyperaemia. Animals with infarct sizes between one third and a half of the left ventricular circumference were taken into account.

### Flow cytometry

A blood sample needed for flow cytometry was taken from the heart into glass vacuum tubes with EDTA anticoagulant by cardiac puncture. One hundred microliters of the whole blood was pre-treated with an Fc receptor-blocking reagent (Miltenyi Biotec GmbH, Bergisch Gladbach, Germany) to prevent nonspecific binding and was incubated for 20 min in the dark at 25°C with 5 μL of anti-CD15s Alexa Fluor 647-conjugate antibodies (Pharmingen, San Diego, CA), 2 μL of phycoerythrin-conjugated antibodies reactive to rat CD11b (BD Pharmingen, San Diego, CA) and 5 μL of mouse antibodies reactive to rat CD44H conjugated with FITC (BD Pharmingen, San Diego, CA). Following the red blood cell lysis with lysis solution (Miltenyi Biotec GmbH, Bergisch Gladbach, Germany), both single and double-colour staining fluorescence was measured on a flow cytometer BD Accuri 6 (BD Biosciences, Belgium). Unstained cell samples were measured and processed as negative controls to set the appropriate regions. Cell acquisition was stopped at 10^6^ cells.

### Data analysis

**Fig 1:**
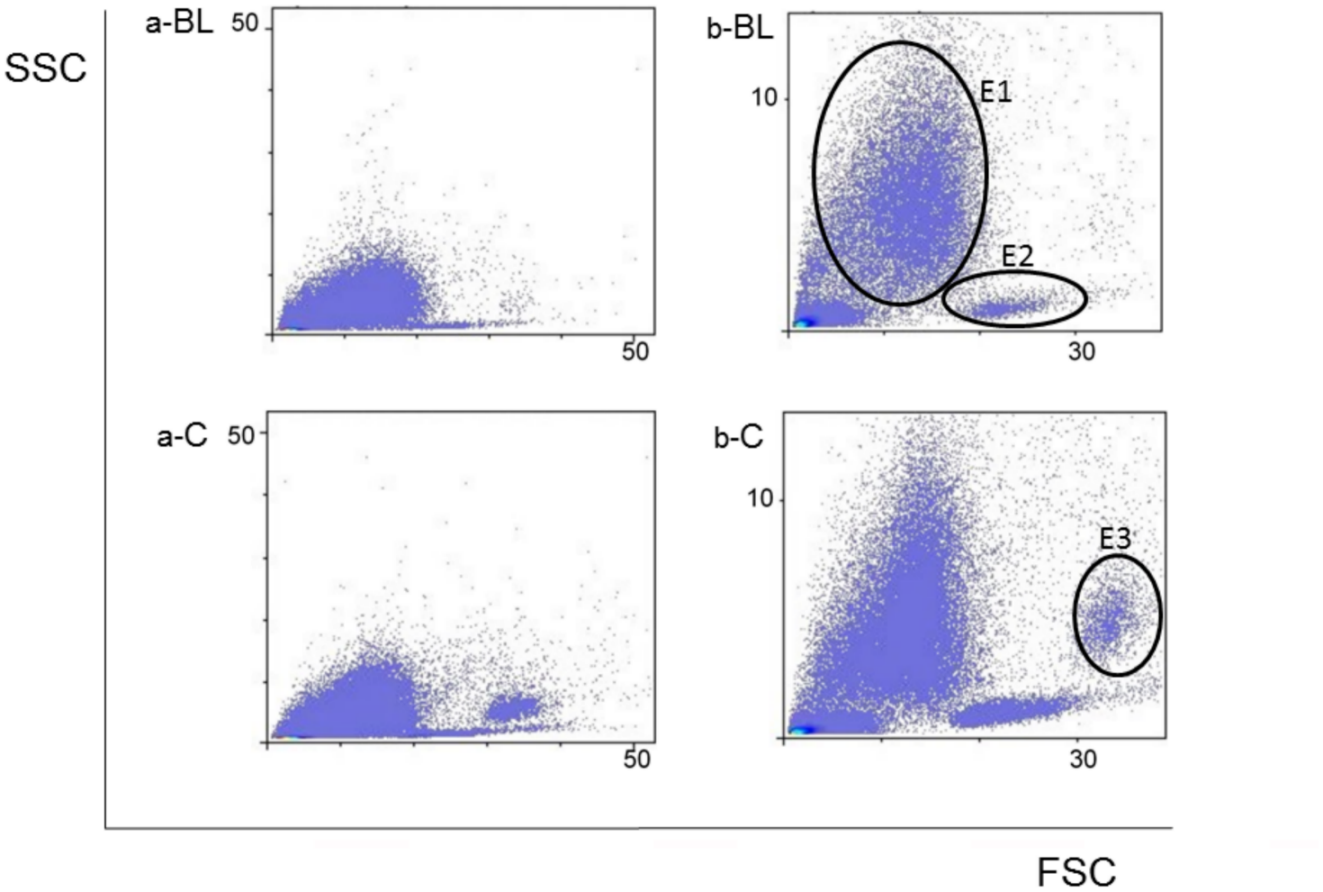
Representative gates for granulocytes (E1), monocyte (E2) and large monocyte (E3) populations from baseline (BL) and control (C) group. Population gates on dot plots a-BL and a-C could not be determined, so they were zoomed and dot plots set “b” was created. The subpopulation of large monocytes appears in control group of animals, subjected to myocardial infarction but not in baseline group.

Data acquired by cytometer were analyzed using the FlowLogic Software (Inivai Technologies, Mentone Victoria, Australia). Leukocyte fluorescence is shown in the forward scatter/side scatter (FSC/SSC) dot plots (Fig 1). FSC parameter indicates cell diameter and SSC indicates cell granularity. The first set of results of flow logic analyses revealed joined leukocyte populations (Fig 1, dot plots a-BL and a-C) giving no possibility to distinguish granulocyte and monocyte populations. Zooming in allowed higher resolution resulting in creation of b-BL and b-C dot plots (Fig 1). Clearly visible granulocyte population was delineated with ellipse E1, while two monocyte subpopulations, with lower and higher FSC/SSC values, were delineated with ellipse E2 and E3, respectively (Fig 1). Two monocyte subpopulations were observed in our study: with lower (called monocytes in this study) and higher forward and side scatter values (called large monocytes in this study) [21]. Expression of adhesion molecules on white blood cells was quantified as fold change in median fluorescence intensity with respect to the baseline group. Since the influence of myocardial infarction to neutrophil and monocyte CD11b expression is already well established [22, 23], we used them (rather than lymphocytes) as the primary focus of our study. The lymphocyte population was mixed with erythrocyte debris that remained after red blood cell lysis. The lymphocytes were not separated from erythrocyte debris by further centrifugation after red blood cell lysis [24], hence they are positioned below granulocytes in the left corner of Fig 1.

### Statistical analysis

For statistical analysis, Mann Whitney test was performed for comparing two groups. The existence of trends among groups was validated by the Test for linear trend. All statistical analysis were performed using Past 3.X software with the significance set at P<0.05 [25]. Sample size was calculated based on Mead’s resource equation.

## Results

### Large monocytes

**Fig 2:**
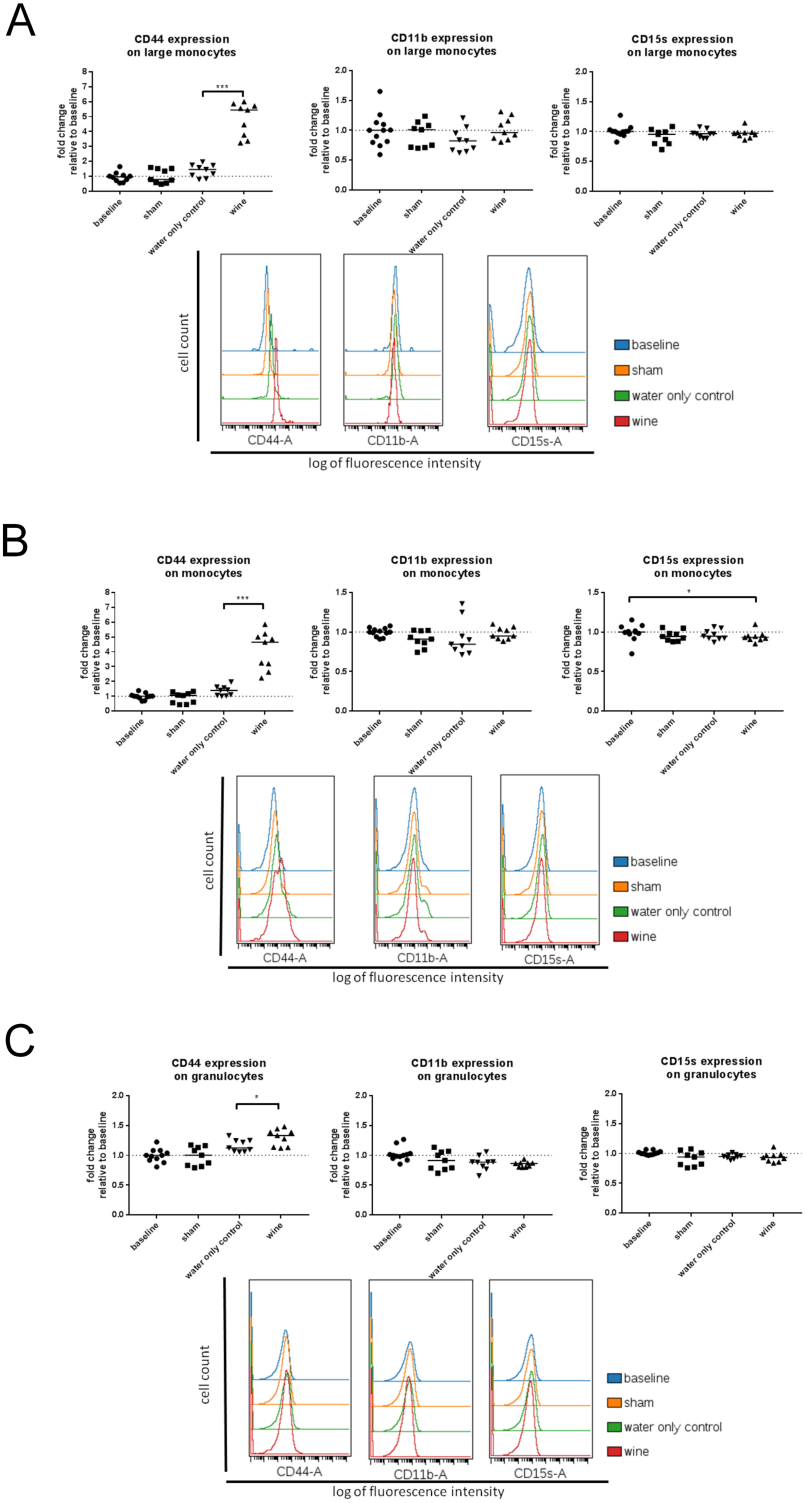
Expression of CD44, CD11b and CD15s on A) large monocytes, B) monocytes and C) granulocytes. Median fluorescence intensity (MFI) of each group is normalised to and presented as fold change from baseline. Data for each subpopulation set is represented in diagrams with median values marked. Mann Whitney test was used for data analyses. Representative fluorescence histograms for each experimental group are provided. *-P<0.05, ***-P<0.001.

Large monocytes from blood of wine drinking animals were characterised by a 3.9-fold (95% CI 2.28 to 4.82, p = 0.0001) increase of CD44 expression, when compared with blood samples from animals drinking water only i.e. control group. Expression of CD44 in control animals did not differ when compared to animals from sham or baseline groups (Fig 2A). Furthermore, comparison of baseline and sham rats did not reveal any difference in CD44 expression on large monocytes. In contrast to CD44, expression levels of CD11b and CD15s were not affected by neither the wine consumption nor by the MI (Fig 2A). Subpopulations of large monocytes, according to their expression of adhesion molecules, showed no differences in percentage representation between experimental groups (Table 1).

**Table 1:** Subpopulations of white blood cells.

Experimental group								
	Baseline		Sham		Control		Wine	
Large Monocytes	Median	IQR	Median	IQR	Median	IQR	Median	IQR
**CD44+ (%)**	98.6	96.93–99.89	99.2	98.01–99.23	97.5	95–99.55	99.4	96.75–99.7
**CD15s+ (%)**	20.5	16.15–35.15	16.77	13.88–35	30.4	25.8–36.05	31.4	23.65–36.05
**CD11b+ (%)**	98.23	97.23–99.57	99.04	98.3–99.6	98	96.75–99.45	99.4	95.95–99.45
**CD44+CD11b+ (%)**	95.98	93.55–98.23	98.16	95.74–98.83	97.7	97.17–98.84	95.54	92.63–97.4
**Monocytes**								
**CD44+ (%)**	64^b^	21.7–67.7	45.83^b^	27.5–54.66	35.2^a^^,^ ^b^	18.85–43	66.7^a^	61.25–71.1
**CD15s+ (%)**	21.92	17.22–36.53	14.17	12.29–38.9	32.9	29.6–39.8	30.9	27.3–34.05
**CD11b+ (%)**	50.1	28.36–54.03	27.11	20.18–54.6	40.5	33.7–58.8	49.9	46.5–57.45
**CD44+CD11b+ (%)**	12.4	9.31–13.1	8.9	7.6–13.26	11.22	9.88–15.74	11.13	9.4–17.73
**Granulocytes**								
**CD44+ (%)**	4.61	1.49–14.97	3.72	2.17–6.22	2.51 ^a^	1.91–4.97	13.29 ^a^	5.66–15
**CD15s+ (%)**	24.21	23.15–29.08	24.86	16.7–32.96	27.35	25.12–29.3	27.57	26.4–29.39
**CD11b+ (%)**	12.94	10.78–15.97	13.53	5.51–18.37	12.72	7.45–15.27	10.24	8.35–10.98
**CD44+CD11b+ (%)**	0.46	0.19–1.04	0.24	0.15–0.58	0.48	0.30–0.62	0.48	0.30–0.62

IQR- interquartile range

^a^- p<0.05 for wine vs. control groups

^b^- p<0.05 for trend between baseline, sham and control group

### Monocytes

White wine consumption caused a 3.2-fold (95% CI 1.569 to 3.86, p<0.0001) increase in CD44 expression on monocytes in comparison to the water drinking controls (Fig 2B). Animals from baseline and sham groups had CD44 expression levels similar to those of controls (Fig 2B). CD11b expression levels on monocytes were similar among all experimental groups (Fig 2B). CD15s expression was slightly decreased (0.06-fold, 95% CI 0.12 to 0.01, p = 0.0323) in wine drinking group when compared to the baseline group. Sham and control groups showed similar trend, but differences failed to reach statistical significance. Regarding percentage representation of CD44+ monocytes, there was a gradual drop in values related to the severity of injury among the baseline, sham and control groups (average drop = 8.75%, 95% CI -17.59 to -0.24, p = 0.0494) (Table 1). In the wine drinking group no drop was observed, and the values remained practically identical to those of the baseline group (Table 1).

### Granulocytes

CD44 expression on granulocytes from wine group was slightly increased (0.2-fold, 95% CI 0.29 to 0.02, p = 0.0203) in comparison to controls (Fig 2C). No differences in CD11b and CD15s expression were found between experimental groups. In the wine group, the percentage of CD44 positivity on granulocytes was highest and significantly differed from controls (Table 1).

## Discussion

The aim of this study was to evaluate the effects of four weeks long consumption of white wine to the expression of granulocyte and monocyte inflammatory markers (CD15s, CD11b and CD44), 24h after the surgically inflicted myocardial infarction.

We focused solely on measurements of humoral inflammatory parameters because of lack of reliable and accurate measurement techniques of the infarct size at the acute stage of infarct healing. In order to establish if our surgical approach to the heart affects the expression of mentioned inflammatory markers, sham animals group was introduced and compared to the baseline animals. No differences in percentage of CD44+ subpopulation or CD44 median fluorescence intensity (MFI) were found between groups. This is an encouraging proof that our original, diaphragmectomy-based approach can be regarded as truly minimally invasive surgical model of MI, because it offers not only an almost bloodless entry to the mediastinum and excellent visibility of the heart but it also prevents triggering measurable changes of neutrophil and monocyte inflammatory markers in circulation.

Animal models of MI indicate that monocyte subsets coordinate the initiation and resolution of cardiac inflammation as well as healing processes after the MI [26]. Immediately after the onset of ischemia, intravital microscopy can detect a rapid leukocyte infiltration to the injured murine myocardium [27, 28]. Coordinated actions of different cell types as well as participation of extracellular matrix, neutrophil and mononuclear cell contribution are needed for the resolution of inflammation following MI [2]. Intravital microscopy of the beating mice heart detected the presence of recruited monocytes within 30 minutes after MI. Monocytes are recruited first from the vascular reservoir and later from the spleen. [27]. In a clinical model of acute MI, blood analyses of patients undergoing transcoronary ablation of septal hypertrophy showed elevated counts of polymorphonuclear neutrophils that appeared within 15 minutes, with a significant increase starting from 2 to 24 hours, while the monocyte number slightly decreased and started to increase approximately 24 h after this procedure [29]. The described leukocyte recruitment dynamics guided us to choose a time window of 24 hours after the MI for analysis of the adhesion molecule expression.

Endothelial selectin binding to CD15s neutrophil glycoantigen mediates the initial tethering of neutrophils to the endothelial cell surface [1]. Normally, CD15s binding to endothelial selectin leads to integrin activation (such as CD11b) [1]. Expression of CD11b enables leukocytes to pass from the blood stream to the tissue through interaction with a cardiomyocyte ICAM-1 [30]. In our study, a tendency of decreasing expression of CD15s on monocytes associated with wine consumption was observed. This is in line with results of Sacanella et al., who observed declined monocyte CD11b and CD15s expression in healthy women, after moderate white wine consumption [31].

The role of neutrophil and monocyte CD44 receptor is crucial after these cells pass the endothelial barrier due to CD44 interaction with hyaluronate in extracellular matrix [5–7]. The lack of CD44 correlates with enhanced and prolonged neutrophil and macrophage infiltration and increased expression of proinflammatory cytokines in the infarcted myocardium [10]. It is well known that MI disrupts myocardial hyaluronate network [10, 32]. Digestion of extracellular matrix and fibrosis of the tissues in the infarcted myocardium are important during first 1 to 2 weeks after MI [33]. Hyaluronate is crucial for structural maintenance of tissue and regulation of inflammation [8]. In addition to its role in regulation of the postinfarction inflammatory response, CD44 has a role in fibrous tissue deposition in the healing infarct and in cardiac remodelling [10]. CD44-mediated interactions may play a direct stimulative role in fibroblast proliferation [34]. Anti-CD44 Ab blocks fibroblast migration on the provisional matrix proteins fibronectin, fibrinogen, and hyaluronate, and induces fibroblast apoptosis [35, 36]. CD44-hyaluronate interactions cause neutrophil apoptosis and shortening of inflammation, which can improve left ventricle function [37]. Augmented dilation of the infarcted heart may be due to the healing defects associated with CD44 deficiency resulting in marked decrease in collagen deposition in the scar [10]. Therefore, CD44-mediated removal of hyaluronate fragments may have crucial influence on suppression of postinfarction inflammation [2, 38]. Knowing this role of CD44 in cardiac repair, we paid a particular attention to analyse CD44+ granulocytes and monocyte subpopulations. Wine drinking caused striking increase in CD44 expression in both monocyte subpopulations. Due to their CD11b expression, almost all (96%) large monocytes from the wine drinking group were CD11b+CD44+ and able to enter the cardiac tissue. The CD44 expression at each large monocyte was fourfold higher in comparison to the water drinking controls. In addition, CD44 expression was threefold higher in ordinary monocytes in wine drinking group compared to the water drinking controls. This is a very specific finding caused by wine consumption.

It was out of the scope of our study to determine which non-alcoholic components of the white wine could be responsible for the observed effects. It should be mentioned, however, that white wine is relatively rich in non-flavonoid compounds, such as phenolic acids (derivatives of hydroxybenzoic and hydroxycinnamic acid) and other phenols such as tyrosol, that have proven and potent biological effects [39, 40].

Neutrophils and monocytes create inflammatory microenvironment that is crucial for the recruitment of cardiac progenitor cells and repair of the injured myocardium [23]. Anti-inflammatory strategies such as depletion of either neutrophils or monocytes proved ineffective in improvement of the cardiac function after MI [23]. Significant effect of wine consumption before MI upon monocyte CD44 expression on the surface of CD44+CD11b+ monocyte subpopulations tempts to continue this investigation. Combined biochemical, histological and cytological approaches during 1–2 week after MI would be useful in elucidating the relationship between wine consumption prior to MI and modulation of the inflammatory microenvironment favourable for the cardiac regeneration.

## Supporting information

S1 File ARRIVE checklist. (PDF)
